# Improving the nutritional evaluation in head neck cancer patients using bioelectrical impedance analysis: Not only the phase angle matters

**DOI:** 10.1002/jcsm.13577

**Published:** 2024-10-24

**Authors:** Aura D. Herrera‐Martínez, Inmaculada Prior‐Sánchez, María Luisa Fernández‐Soto, María García‐Olivares, Cristina Novo‐Rodríguez, María González‐Pacheco, María José Martínez‐Ramirez, Alba Carmona‐Llanos, Andrés Jiménez‐Sánchez, Concepción Muñoz‐Jiménez, Fátima Torres‐Flores, Rocío Fernández‐Jiménez, Hatim Boughanem, María Carmen del Galindo‐Gallardo, Luis Miguel Luengo‐Pérez, María Josefa Molina‐Puerta, José Manuel García‐Almeida

**Affiliations:** ^1^ Maimonides Institute for Biomedical Research of Cordoba (IMIBIC) Córdoba Spain; ^2^ Endocrinology and Nutrition Service Reina Sofia University Hospital Córdoba Spain; ^3^ Endocrinology and Nutrition Service Jaen University Hospital Jaen Spain; ^4^ Endocrinology and Nutrition Service, San Cecilio University Hospital, Instituto Biosanitario de Granada University of Granada Granada Spain; ^5^ Endocrinology and Nutrition Service Instituto de Investigación Biomédica de Málaga (IBIMA) Regional University Hospital Málaga Spain; ^6^ Endocrinology and Nutrition Service Virgen de las Nieves University Hospital Granada Spain; ^7^ Endocrinology and Nutrition Service, Instituto de Investigación e Innovación Biomédica de Cádiz (INIBICA) Puerta del Mar University Hospital Cádiz Spain; ^8^ Endocrinology and Nutrition Service, Instituto de Investigación e Innovación Biomédica de Cádiz (INiBICA) Jerez de la Frontera University Hospital Cadiz Spain; ^9^ Endocrinology and Nutrition Service, Virgen del Rocío University Hospital Instituto de Biomedicina de Sevilla (IBIS) Seville Spain; ^10^ Endocrinology and Nutrition Service Valme University Hospital Seville Spain; ^11^ Endocrinology and Nutrition Service, Virgen de la Victoria University Hospital Instituto de Investigación Biomédica de Málaga (IBIMA) Málaga Spain; ^12^ Endocrinology and Nutrition Service Macarena University Hospital Seville Spain; ^13^ Fundación pública para la gestión de Salud en Sevilla (FISEVI) Madrid Spain; ^14^ Department of Biomedical Sciences, Endocrinology and Nutrition Service, Badajoz University Hospital Universidad de Extremadura Badajoz Spain

**Keywords:** Bioelectrical impedance analysis, Head neck cancer, Malnutrition, Sarcopenia

## Abstract

**Background:**

Malnutrition and sarcopenia are highly prevalent in patients with head neck cancer (HNC). An accurate early diagnosis is necessary for starting nutritional support, as both are clearly associated with clinical outcomes and mortality. We aimed to evaluate the applicability and accuracy of body composition analysis using electrical bioimpedance vectorial analysis (BIVA) for diagnosing malnutrition and sarcopenia in patients with HNC cancer undergoing systemic treatment with chemotherapy or radiotherapy.

**Methods:**

Cross‐sectional, observational study that included 509 HNC patients. A comprehensive nutritional evaluation that included BIVA was performed.

**Results:**

The prevalence of malnutrition was higher in patients that received treatment with chemotherapy (59.2% vs. 40.8%, *P* < 0.001); increased mortality was observed in malnourished patients (33.3% vs. 20.1%; *P* < 0.001); ECOG status (1–4) was also worse in malnourished patients (59.2% vs. 22.8% *P* < 0.001). Body cell mass (BCM) and fat mass were the most significantly associated parameters with malnutrition [OR 0.88 (0.84–0.93) and 0.98 (0.95–1.01), respectively]; BCM and fat free mass index (FFMI) were associated with several aspects including (1) the patient‐generated subjective global assessment [OR 0.93 (0.84–0.98) and 0.86 (0.76–0.97), respectively], (2) the presence of sarcopenia [OR 0.81 (0.76–0.87) and 0.78 (0.66–0.92), respectively]. A BCM index (BCMI) < 7.8 in combination with other parameters including FFMI and BCM accurately predicted patients with malnutrition [accuracy 95% CI: 0.803 (0.763–0.839); kappa index: 0.486; AUC: 0.618 (*P* < 0.01)]. A BCMI cutoff of 7.6 was enough for identifying males with malnutrition (*P* < 0.001), while it should be combined with other parameters in females.

**Conclusions:**

Body composition parameters determined by BIVA accurately identify patients with HNC and malnutrition. Phase angle, but other parameters including BCMI, FFMI and BCM provide significant information about nutritional status in patients with HNC.

## Introduction

Head neck cancer (HNC) is the seventh most common cancer worldwide. In Europe its prevalence accounts for approximately 21.8 per 100 000, with mortality rates approximately 15.6 per 100 000.^1^ In the United States, is the fourth cancer in prevalence, and it is estimated that 15 400 deaths (11 210 men and 4190 women) will occur in 2023.^2^

Affected patients can have long‐term consequences including decreased food intake, tooth loss, mucositis, and dysphagia, which in turn affect their nutritional condition; additionally, local effects of the tumour, and the tumour itself can produce weight and muscle mass loss.^3^ Additionally, clinical and surgical management of these patients frequently affects the baseline nutritional status of the patient.^4^

In this context, malnutrition is frequently observed, its prevalence ranges 20–74% depending on the series and the diagnosis method.^5^, ^6^, ^7^, ^8^, ^9^ Some studies have reported malnutrition using body mass index (BMI), albumin serum levels, skin fold thickness or their combination.^10^ But, as the publication of the new diagnosis criteria for malnutrition in 2018 by the Global Leadership Initiative on Malnutrition (GLIM),^11^ the re‐evaluation of the prevalence and effects of malnutrition should be assessed.

Moreover, recent studies have highlighted the use of body composition variables determined by electrical bioimpedance vectorial analysis (BIVA) as they can be related to clinical outcomes in several types of cancer.^12^ Despite this, specific cutoff points for malnutrition or sarcopenia are not routinely used in the clinical practice.

In this context, we present a multicentre study, in which a nutritional evaluation including BIVA was performed in a large cohort of patients with HNC. We aimed to evaluate the reliability of this comprehensive nutritional evaluation in patients with HNC and to determine the applicability of specific cutoff values in several body composition parameters, determined by BIVA, for identifying patients with HNC and malnutrition.

## Materials and methods

### Study design

This is a multicenter, a cross‐sectional observation study, in which 509 patients with HNC of different stages were included. Patients were evaluated at the Endocrinology and Nutrition Unit of 11 hospitals in Spain. This study was approved by the Ethics Committee of the Reina Sofia University Hospital (Cordoba, Spain; reference number 5006), and was conducted in accordance with the Declaration of Helsinki and according to national and international guidelines. A written informed consent was signed by every individual before inclusion into the study, this consent included the initial evaluation and long‐term follow‐up including survival data. All patients received information before the inclusion and only if accepted to participate, were included. A confirmed histological diagnosis according to the current classification systems was required to participate^13^; patients with life expectancy of <3 months were not included.

### Nutritional evaluation

This study includes a comprehensive nutritional evaluation, which has been reported by our group in previous studies.^12^, ^14^, ^15^ Briefly, nutritional evaluation was performed before or during the first 2 weeks of systemic treatment (chemotherapy, RT, or their combination). Body‐composition analysis was performed using a 50 kHz phase‐sensitive impedance analyser (BIA 101 Whole Body Bioimpedance Vector Analyser, AKERN, Florence, Italy) that delivers 800 μA using tetrapolar electrodes positioned on the right hand and foot. All BIVA measurements were obtained with the patient in a supine position after 5 min of rest. BIVA emphasizes the position of the impedance vector, derived from resistance (R) and reactance (Xc) values generated from a sex‐specific healthy reference population.^16^ BIA measurements of patients were standardized for sex and age using data from healthy Italian adults.^17^ Phase angle (PA) is expressed in degrees as arctan (Xc/R) × (180°/***π***).^17^ Individual standardized PA value (SPA) was determined from the sex‐ and age‐matched reference population value.^18^ The technical accuracy of the BIA instrument was daily assessed using a precision circuit supplied by the BIA device manufacturer (AKERN, Florence, Italy). All measured R and Xc values were consistently ±1 Ω of the 385 Ohm reference value. In vivo reproducibility of the BIA measurements was also determined, with coefficients of variation (CV) of 1 2% for R and Xc.

BIA provides bioimpedance‐derived parameters for hydration [fluid percentage within the fat‐free mass (FFM) values] and nutrition status (creatine excretion rate in mg/kg/24 h obtained from body cell mass (BCM) values. All bioimpedance data from BIA were categorized as fat free mass (FFM: kg) and FFM index (FFMI: %); fat mass (FM: kg) and FM index (FMI: %); muscle mass (MM: kg); skeletal muscle mass (SMM: kg), appendicular skeletal muscle mass (ASMM: kg) and SM index (SMI: %); intracellular water (ICW: kg); total body water (TBW: kg); and BCM (kg). Height measurements were taken using a 2 mm sensitivity laser height rod.

### Functional evaluation

Handgrip strength (HGS) was measured using a JAMAR® hand dynamometer (Asimow Engineering Co., Los Angeles, CA, USA). HGS was measured in a seated position with the elbow flexed at 90 degrees in the dominant hand. The median value of three maximal isometric contractions was used. Additionally, the timed up and go test (TUG) was performed, in which a seated patient gets up, walks 3 m, turns around, walks another 3 m, and sits back down, the used time is measured in seconds; a cutoff point of 10.85 s was used for defining sarcopenia.^19^

#### Assessment of malnutrition and sarcopenia

Malnutrition was diagnosed according to the GLIM criteria.^11^ Patients with moderate malnutrition presented with BMI < 20 kg/m^2^ (age <70) or <22 kg/m^2^ (age ≥70), weight loss of 5–10% or FFMI <15 and 17 for females and males, respectively. Severe malnutrition was diagnosed in cases with weight loss >10%, BMI < 18.5 kg/m^2^ (age <70) or <20 kg/m^2^ (age ≥70).^11^

In this study, sarcopenia was defined using the HGS, with a cutoff point of <27 kg in men and <16 kg in women; according to the European Working Group on Sarcopenia in Older People's diagnostic criteria, it is recommended to use two criteria: low muscle mass and low muscle function (strength or performance),^20^ as there are no specific cutoff points of muscle mass for this population, we only used the muscle function criterion. Additionally, the Patient‐Generated Subjective Global Assessment (PG‐SGA) was performed; according to this, patients were classified as well‐nourished, moderately malnourished; or severely malnourished.

Additionally, patients were categorized according to their BMI in four groups (<22, 22–25, 25–50, and >30 kg/m^2^), and body composition parameters determined by BIVA were compared according to the percentage of weight loss (<5%, 5–10%, and >10%).

Biochemical analysis was automatically performed in the biochemical unit of every hospital, it included serum measurements of albumin, prealbumin, total cholesterol, glucose, urea, creatinine, thyrotropin hormone, and HbA1c.

#### Statistical analysis

Results are presented as median ± interquartile range for continuous variables (except for age and initial body weight, which are presented in mean ± standard deviation) and as percentages for categorical variables. Between‐groups comparisons were performed using Mann–Whitney *U* and Wilcoxon test (non‐parametric data). Spearman correlation coefficients between variables were performed. The odds ratio (OR) and 95% confidence intervals (CIs) were obtained using logistic regression analysis. The evaluation of the predictive property of nutritional variables were based on the receiver operating characteristic (ROC) curve and area under the curve (AUC). Decision trees were conducted using *rpart* package and random forest was performed using *Randomforest* package. Age, sex, and BMI were the variables that mostly affected BIA parameters, especially muscle mass related parameters; thus, they were selected and included in the predictive model. Specifically, a BMI cutoff point of 25 kg/m^2^ was used to differentiate normal weight and overweight in patients with sarcopenia; a cutoff point of 20 kg/m^2^ in the VSG analysis and a cutoff of 20 or 22 kg/m^2^ in patients <70 or ≥70 years old. Analyses and graphic representation were performed using Rv.3.5.1 software (Integrated Development for R. RStudio, PBC, Boston, MA, USA), and the significance *P* value was set at *P* < 0.05.

## Results

### General characteristics of the cohort

A total of 509 patients were included, most patients were male (76.8%) with a mean age at diagnosis of 63.9 years old. One fifth of patients had a stage II of disease at diagnosis (20.6%), followed by stage IVB (31%) and stage IVA (13.9%). All patients received RT and 45.6% also received chemotherapy. According to the Eastern Cooperative Oncologic Group (ECOG) performance scale, more than 90% of patients presented with ECOG 0 and 1 at diagnosis (47.6 and 43.9%, respectively), additionally, patients presented with body weight loss of 6.21% at that time (Table 1).

**Table 1:** Baseline characteristics of the population of study depending on the presence of malnutrition according to the GLIM criteria

	All	Well‐nourished	Malnourished	*P* value
	*N* = 509	*N* = 352	*N* = 157	
Demographic variables				
Age (years)	63.9 (10.1)	63.3 (9.73)	65.1 (10.8)	0.083
Sex (males/females)	391/109	280/70	111/39	0.170
BMI (kg/m^2^)	25.2 (4.52)	26.0 (4.38)	23.5 (4.39)	<0.001***
Weight (kg)	70.5 (14.8)	73.0 (14.3)	64.7 (14.5)	<0.001***
Weight loss (%)	6.21 (9.12)	3.53 (8.30)	12.0 (8.10)	<0.001***
Clinicopathological variables				
Cancer stage				0.356
I	34 (7.07%)	24 (7.21%)	10 (6.76%)	
II	40 (8.32%)	32 (9.61%)	8 (5.41%)	
III	99 (20.6%)	70 (21.0%)	29 (19.6%)	
IVA	67 (13.9%)	46 (13.8%)	21 (14.2%)	
IVB	149 (31.0%)	94 (28.2%)	55 (37.2%)	
IVC	92 (19.1%)	67 (20.1%)	25 (16.9%)	
Mortality				0.001**
No	310 (75.1%)	231 (79.9%)	79 (63.7%)	
Yes	103 (24.9%)	58 (20.1%)	45 (36.3%)	
Chemotherapy				0.174
No	232 (45.6%)	168 (47.7%)	64 (40.8%)	
Yes	277 (54.4%)	184 (52.3%)	93 (59.2%)	
ECOG				<0.001***
0	217 (47.6%)	184 (59.2%)	33 (22.8%)	
1	200 (43.9%)	114 (36.7%)	86 (59.3%)	
2	32 (7.02%)	12 (3.86%)	20 (13.8%)	
3	5 (1.10%)	1 (0.32%)	4 (2.76%)	
4	2 (0.44%)	0 (0.00%)	2 (1.38%)	

Data are expressed as mean ± standard deviations or percentage. Groups were divided by well‐nourished, and malnourished according to the GLIM criteria. Asterisk indicates significant difference between groups, according to the Mann–Whitney test (chi squared test was used for variables expressed as percentage.

BMI, body mass index; ECOG, Eastern Cooperative Oncologic Group performance scale.

* *P* < 0.05.

** *P* < 0.01.

*** *P* < 0.001.

According to the GLIM criteria, 30.8% (*n* = 157) presented malnutrition at diagnosis. Patients with malnutrition presented with lower body weight, BMI and a higher rate of body weight loss when compared with patients without malnutrition (*P* < 0.001). Remarkably, a similar distribution of cancer stage at diagnosis and treatment with chemotherapy was observed in patients with and without malnutrition. A higher proportion of malnourished patients presented with ECOG1 (59.3% vs. 36.7%), while ECOG 0 was more common in well‐nourished patients (59.2% vs. 22.8%; *P* < 0.001). Mortality rate was higher in patients with malnutrition at diagnosis (36.3%) when compared with well‐nourished patients at diagnosis (20.1%; *P* < 0.001). Specific comparisons and characteristics of the general cohort are depicted in Table 1.

### Nutritional evaluation using bioelectrical impedance vector analysis

Body composition using BIVA revealed that patients presented with a median PA of 5.2 (standardized of −0.6), FFMI of 18.4% (16.6; 20.0), FMI of 6.6% (4.60; 8.30), BCMI of 9% (7.80; 10.5) and SMI of 8.9 (7.70; 9.70). Rz was significantly higher in patients with malnutrition; as expected, PA, SPA, BCM, FM, FFMI, FMI, BCMI, and SMI were significantly decreased in malnourished patients (*P* < 0.001). Additionally, MM, SMM, ASMM, and FFM were significantly lower in patients with malnutrition; furthermore, TBW, ECW, and ICW were also significantly decreased in malnourished patients (*P* < 0.001). In contrast, the sodium potassium ratio (NAK) was significantly increased in patients with malnutrition (*P* < 0.001; Table 2).

**Table 2:** Nutritional evaluation according to the presence of malnutrition, defined by GLIM criteria

Variables	All	Well‐nourished	Malnourished	*P* value
	*N* = 500	*N* = 352	*N* = 150	
BIVA				
Xc (Ω/m)	50.0 [43.0; 56.0]	50.0 [43.7; 56.0]	49.0 [42.5; 55.5]	0.539
Rz (Ω/m)	540 [490; 610]	525 [479; 587]	579 [516; 651]	<0.001
PA (°)	5.20 [4.60; 5.80]	5.30 [4.80; 5.90]	4.80 [4.20; 5.40]	<0.001
SPA	−0.60 [−1.33; 0.30]	−0.40 [−1.20; 0.42]	−0.90 [−1.80; −0.05]	<0.001
BCM (kg)	25.6 [20.8; 30.0]	27.1 [22.2; 30.8]	21.4 [18.2; 27.3]	<0.001
FM (kg)	18.1 [13.0; 23.9]	19.2 [14.1; 24.9]	15.9 [10.8; 22.0]	<0.001
FFMI (%)	18.4 [16.6; 20.0]	18.9 [17.2; 20.4]	17.0 [15.6; 19.0]	<0.001
FMI (%)	6.60 [4.60; 8.30]	6.80 [5.10; 8.60]	5.60 [4.00; 7.70]	<0.001
BCMI (%)	9.00 [7.80; 10.5]	9.40 [8.20; 10.9]	7.90 [6.90; 9.30]	<0.001
SMI (cm^2^/m^2^)	8.90 [7.70; 9.70]	9.10 [8.20; 9.90]	8.20 [7.10; 9.20]	<0.001
Muscle mass variables				
MM (kg)	25.8 [21.9; 29.2]	26.4 [22.9; 29.8]	24.1 [20.4; 27.6]	<0.001
SMM (kg)	25.9 [21.9; 29.2]	26.5 [22.9; 29.8]	24.1 [20.4; 27.6]	<0.001
ASMM (kg)	19.6 [16.6; 22.3]	20.4 [17.7; 22.6]	17.6 [15.2; 20.9]	<0.001
FFM (kg)	51.7 [45.0; 58.2]	53.5 [47.3; 59.2]	48.3 [41.1; 54.1]	<0.001
Water content variables				
TBW (kg)	38.1 [32.9; 42.9]	39.2 [34.5; 43.5]	35.9 [30.0; 40.2]	<0.001
ECW (kg)	18.8 [16.2; 21.1]	19.0 [16.8; 21.1]	18.3 [15.6; 21.0]	0.138
ICW (kg)	50.1 [46.7; 53.5]	51.1 [47.8; 54.0]	48.0 [43.9; 51.4]	<0.001
NAK	1.10 [1.00; 1.23]	1.10 [1.00; 1.20]	1.15 [1.00; 1.30]	<0.001
Metabolism and nutrition				
Basal metabolism (kcal)	1490 [1353; 1617]	1539 [1391; 1640]	1372 [1278; 1530]	<0.001
Hydration (%)	73.4 [73.1; 73.6]	73.4 [73.2; 73.6]	73.4 [73.1; 73.6]	0.615
Nutrition	765 [634; 906]	822 [687; 929]	655 [566; 807]	<0.001
Biochemical variables				
Total cholesterol (mg/dL)	184 [158; 215]	185 [159; 214]	181 [157; 215]	0.371
Proteins (g/dL)	7.00 [6.60; 7.40]	7.00 [6.70; 7.30]	7.00 [6.53; 7.40]	0.475
Albumin (g/dL)	4.00 [3.70; 4.30]	4.10 [3.80; 4.40]	3.80 [3.50; 4.10]	<0.001
Pre‐albumin (mg/dL)	24.3 [20.0; 29.2]	25.4 [20.8; 30.8]	22.5 [18.6; 28.1]	0.025
Functional measurement				
HGS max (kg)	32.5 [25.0; 40.0]	34.8 [28.0; 42.0]	29.0 [20.0; 36.0]	<0.001
HGS mean (kg)	31.0 [24.0; 38.3]	32.7 [26.0; 39.6]	27.7 [20.0; 34.0]	<0.001
TUG (s)	7.50 [6.00; 10.0]	7.41 [5.90; 9.82]	8.00 [6.38; 10.0]	0.042

Data are expressed as median ± interquartile range or percentage. Groups were divided by well‐nourished, and malnourished according to the GLIM criteria. Asterisk indicates significant difference between groups, according to the Mann–Whitney test (chi squared test was used for variables expressed as percentage.

ASMM, appendicular skeletal muscle mass; BCM, body cell mass; BCMI, body cell mass index; BIVA, bioelectrical impedance vector analysis; BM, basal metabolism; BMI, body mass index; ECW, extracellular cell water; FFM, fat free mass; FFMI, fat free mass index; FM, fat mass; FMI, fat mass index; HGS, handgrip strength; MM, mass muscle; NAK, sodium and potassium ratio; PA, phase angle; Rz, resistance; SPA, standardized phase angle; SSM, skeletal muscle mass; TBW, total body water; TUG, up and go test; Xc, reactance.

* *P* < 0.05.

** *P* < 0.01.

*** *P* < 0.001.

Additionally, patients were categorized according to their BMI in four groups (<22, 22–25, 25–50, and >30 kg/m^2^) and compared according to the percentage of weight loss (<5%, 5–10% and >10%). We observed that patients with BMI < 22 kg/m^2^ presented significant differences in PA, BCM, BCMI, ASMM, FFM, TBW, ICW, NAK ratio, metabolism and nutrition (Figure S1). When the BMI comprised 22–25 kg/m^2^ differences included the same parameters and additionally SMI, MM, SMM, and FFM (Table S2). When patients presented with BMI 22–25 kg/m^2^ between group significant differences only included Xc, PA, SPA, BCM, ICW, and nutrition (Table S3). Finally, when patients with BMI > 30 kg/m^2^ were analysed, the only parameters that presented body composition differences were PA, FFMI, BCMI, SMI, ICW, and nutrition (Table S4).

### Nutritional evaluation using functional tests and biochemical variables

Evaluated patients presented a maximal hand grip strength of 32.5 kg (25.0; 40.0) and median hand grip strength of 31 kg (24.0; 38.3), while TUG was performed in a median time of 7.5 (6–10) seconds. Patients with malnutrition had decreased maximal and median HGS (29 and 27.7 kg, respectively) compared with patients without malnutrition (34.8–32.7 kg, respectively; *P* < 0.001). Similarly, patients with malnutrition required more time for completing the TUG (*P* = 0.042; Table 2).

Biochemical nutritional parameters revealed that malnourished patients presented with lower serum albumin and prealbumin levels (3.8 g/dL and 22.5 mg/dL) compared with well‐nourished patients (4.10 g/dL and 25.4 mg/dL; *P* < 0.001 and *P* < 0.025; Table 2).

### Usefulness of bioelectrical impedance vector analysis for determining malnutrition and sarcopenia

Age‐, sex‐ and BMI‐adjusted body composition parameters (determined by BIVA) were associated malnutrition (according to the GLIM criteria), sarcopenia and PG‐SGA. Specifically, PA [OR 0.54 (0.40–0.71)], SPA [OR 0.72 (0.59–0.88)], muscle mass parameters [[BCM [OR 0.88 (0.84–0.93)] and BCMI [OR 0.66 (0.56–0.76)]], adipose tissue variables [[FM [OR 0.98 (0.95–1.01)] and FMI [OR 0.94 (0.86–1.03)]] and muscle quality parameters [[FFMI [OR 0.76 (0.66–0.85)] and SMI [OR0.60 (0.48–0.75)]] were associated with malnutrition (Table 3). When PG‐SGA was evaluated, only muscle mass variables and muscle quality parameters were significantly associated, specifically BCM [OR 0.93 (0.84–0.98)], BCMI [OR 0.80 (0.69–0.93)], FFMI [OR 0.86 (0.76–0.97)], and SMI [OR 0.74 (0.58–0.92)]. Finally, PA, SPA, BCM, BCMI, FFMI, and SMI, but not FM or FMI were significantly associated with sarcopenia in patients with HNC (Table 3).

**Table 3:** Multiple logistic regression of nutritional assessment methods and the risk of malnutrition and sarcopenia in patients with head and neck cancer

Variables	GLIM	PG‐SGA	Sarcopenia
	OR (95% CI)	OR (95% CI)	OR (95% CI)
Phase angle			
PA	0.54 (0.40–0.71)***	0.75 (0.56–1.01)	0.47 (0.33–0.66)***
SPA	0.72 (0.59–0.88)**	0.87 (0.69–1.10)	0.70 (0.55–0.89)**
Muscle mass			
BCM	0.88 (0.84–0.93)***	0.93 (0.84–0.98)**	0.81 (0.76–0.87)***
BCMI	0.66 (0.56–0.76)***	0.80 (0.69–0.93)**	0.70 (0.57–0.82)***
FFMI	0.76 (0.66–0.85)***	0.86 (0.76–0.97)*	0.78 (0.66–0.92)**
SMI	0.60 (0.48–0.75)***	0.74 (0.58–0.92)**	0.71 (0.54–0.92)*
Adipose tissue			
FM	0.98 (0.95–1.01)	0.97 (0.94–1.01)	0.99 (0.94–1.03)
FMI	0.94 (0.86–1.03)	0.91 (0.82–1.00)	1.07 (0.93–1.23)

Multiple logistic regression of GLIM (well‐nourished vs. malnourished), PG‐SGA (A vs. B + C) and sarcopenia (absence vs. presence of sarcopenia) and its relationship with nutritional assessment methods. All variables were adjusted for age, sex, and BMI. To test the effect of malnutrition of the association with nutritional ultrasound, in the case of the GLIM, the cut‐off of BMI was BMI < 22 in those patients equal of >70 years old, and a BMI < 20 for those patients aged below than 70 years old. For the PG‐SGA, BMI cut‐off was set in those patients that had a BMI lower than 20. In the case of sarcopenia, the cut‐off of the BMI was set in those patients that had a BMI lower than 25.

BCM, body cell mass; BCMI, BCM index; FFM, fat‐free mass; FFMI, FFM index; OR, odds ratio; FM, fat mass; PA, phase angle; PG‐SGA, Patient‐Generated Subjective Global Assessment; SMI, skeletal mass index; SPA, standardized PA.

* *P* < 0.05.

** *P* < 0.01.

*** *P* < 0.001.

### Cutoff values for predicting malnutrition in head neck cancer patients

BCMI was the parameter with the higher AUC for predicting malnutrition, with a cutoff value of 8 (sensitivity of 82% and a specificity of 52%; *P* < 0.001; Table 4), other muscle mass parameters also were useful for predicting malnutrition, specifically BCM (cutoff value: 21.2; AUC 0.676; *P* < 0.001), FFMI (cutoff value: 17.1; AUC 0.68; *P* < 0.001), and SMI (cutoff value: 8.6; AUC 0.671; *P* < 0.001). The PA had a higher AUC (cutoff value: 4.9; AUC: 0.628; *P* = 0.002) than the SPA (cutoff value: −0.6; AUC 0.571; *P* = 0.09). Cutoff values for FM (17.6) and FMI (6) had an AUC of 0.61 for predicting malnutrition (*P* < 0.05). Significant cutoff values, their AUC, sensitivity and specificity are depicted in Table 4, sex‐specific differences are depicted in Table S5.

**Table 4:** Predictive value of nutritional assessment methods on malnutrition in patients with head and neck cancer

Variables	AUC	Cut‐off	Sensitivity	Specificity	*P* value
Phase angle					
PA	0.628	4.9	71%	67%	0.002**
SPA	0.571	−0.6	60%	61%	0.09
Muscle mass					
BCM	0.676	21.2	83%	70%	<0.001***
BCMI	0.700	8.0	82%	52%	<0.001***
FFMI	0.685	17.1	80%	50%	<0.001***
SMI	0.671	8.6	67%	60%	<0.001***
Fat mass					
FM	0.611	17.6	60%	61%	0.02*
FMI	0.610	6.0	66%	57%	0.014*

Receiver operating characteristic (ROC) and area under curve (AUC) for the nutritional assessment methods to predict malnutrition in patients with head and neck cancer. Cut‐off without adjusting for variables.

AUC, area under curve; BCM, body cell mass; BCMI, BCM index; FFMI, fat‐free mass index; FM, fat mass; FMI, FM index; PA, phase angle; SMI, skeletal muscle index; SPA, standardized PA.

### Correlations between body composition parameters determined by bioelectrical impedance vector analysis and other nutritional parameters

**Figure 1:**
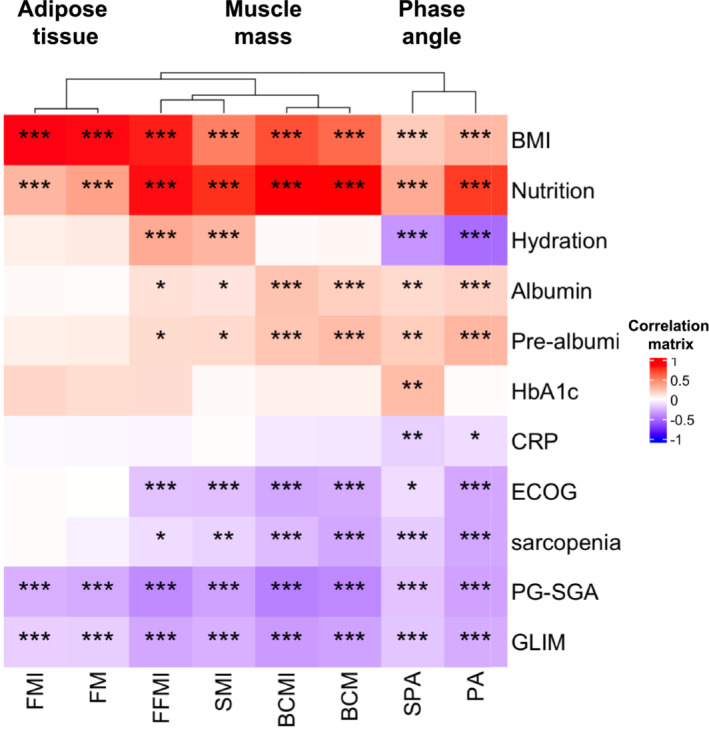
Significant correlations between body composition parameters assessed by BIVA and ultrasound nutritional evaluation, biochemical nutritional parameters, ECOG, sarcopenia, PG‐SGA and malnutrition.

Regarding biochemical parameters, albumin and prealbumin positively correlated with muscle‐mass related parameters (FFMI, SMI, BCMI, and BCM), PA and SPA; additionally, C‐reactive protein (C‐RP) negatively correlated with PA and SPA (Figure 1).

The ECOG was also strongly and negatively correlated with muscle‐related nutritional parameters (SMI, BCM, FFMI, and BCMI), PA, and SPA. As expected, sarcopenia was negatively correlated only with muscle mass parameters (BCMI and BCM), muscle quality parameters (FFMI and SMI), PA, and SPA. Significantly, there was a strong negative correlation between PG‐SGA and malnutrition (assessed by GLIM criteria) with muscle‐related parameters (FFMI, SMI, BCMI, and BCM), fat mass‐related parameters (FMI and FM), PA and SPA in HNC patients (*P* < 0.001; Figure 1).

Some differences were observed when patients were separately analysed by sex. Specifically, in males, albumin and prealbumin levels only positively correlated with BCMI, BCM, PA and SPA and C‐RP negatively correlated with BCMI, BCM, and SPA (Figure S1). In contrast, in female, albumin and prealbumin only correlated positively with PA, and SPA; additionally, sarcopenia negatively correlated with BCM and PA; while the ECOG negatively correlated only with FFMI, SMI, BCMI, and BCM; finally, malnutrition did not correlate with FM of FMI in women (Figure S2).

### Clinical algorithm for evaluating malnutrition using bioelectrical impedance vector analysis in patients with head neck cancer

**Figure 2:**
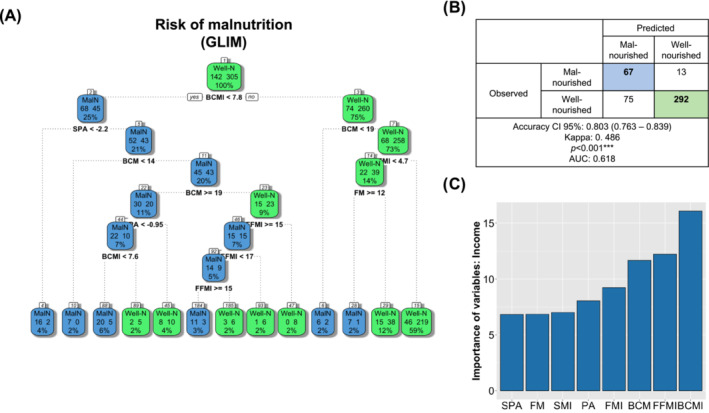
(A) Clinical algorithm for predicting malnutrition in HNC patients using body composition parameters determined by BIVA; (B) accuracy of the clinical model; (C) significance of the included variables in the model.

As previously mentioned, in the whole cohort, BCMI was an useful parameter for evaluating malnutrition. Based on this, we performed a clinical algorithm for accurately evaluating patients with and without malnutrition (Figure 2). Specifically, an initial cutoff value of 7.8 should be used, its combination with a SPA <−2.2 accurately identified patients with malnutrition. If the SPA is >−2.2 but the BCM < 14, malnutrition is also diagnosed. Finally, if BCM ranges 14–19, SPA should be evaluated, if SPA <−0.95 and BCMI <7.6, malnutrition should be diagnosed; in contrast, if BCMI>7.6 or the SPA >−0.95, patients are well‐nourished (Figure 2A). In contrast, when the BCMI is >7.8 but BCM is <19, patients are also malnourished; if BCM is >19, FMI should be evaluated, if FMI < 4.7 but SMI < 4.7 and FM < 12; but if FM ≥ 12 patients should be also classified as well‐nourished (Figure 2A). This model can predict malnutrition with and accuracy 95% CI of 0.803 (0.763–0.839), a kappa index 0.486 and an AUC of 0.618 (*P* < 0.01; Figure 2B). BCMI, FFMI, and BCM were the most relevant variables considered in this model (Figure 2C).

Remarkably, in males, only a BCMI cutoff value of 7.6 accurately identifies patients with malnutrition (Figure S3A), with an accuracy 95% CI 0.757 (0.709–0.801), a kappa index 0.340; and an AUC 0.618 (*P* < 0.001; Figure S3B), in this model, BCMI and FFMI were the most significant parameters to be considered (Figure S3C).

In contrast, in females, a BCM < 14 identified patients with malnutrition; if BCM is ranges 14–19, it should be used in combination with FFMI and FM, specifically a FFMI < 15 and a FM < 19 is observed in patients with malnutrition. Additionally, a BCM > 19 but with a FFMI < 16 also identifies patients with malnutrition (Figure S3D). This model had a kappa index of 0.638 and an AUC of 0.657 (*P* < 0.001; Figure S3E); BCM was the most relevant variable to consider in this model for female patients with HNC (Figure S3F).

## Discussion

Malnutrition can affect about 50% of HNC patients at diagnosis and almost 90% during active treatment.^21^ In this context, it is essential to perform an early and appropriate nutritional evaluation, which can detect patients at risk for malnutrition, in order to initiate early nutrition support for improving treatment adherence, tolerance and avoiding treatment interruptions.^3^ Nutritional evaluation should comprise a comprehensive evaluation, including body composition measurements. Body composition influences the efficacy and toxicity of systemic treatment, it also affects patient functional status, surgical complication rates, length of hospital stay and overall survival.^22^ It has been suggested that about 70% of weight loss in HNC patients corresponds to lean mass,^22^ this loss results in impaired muscle strength, declined physical activity and functional performance, it also increases the risk of recurrence and mortality.^23^ Furthermore, lean mass, which main component is SMM, has been negatively associated with BMI, reflecting that some patients might present with decreased SMM despite normal weight or overweight, which is known as sarcopenic obesity.^24^ In this study, the median values revealed an overweighted population with slightly decreased SMM. Thus, fat mass, muscle mass and functionality should be evaluated using different techniques,^12^ especially if they provide complimentary information and are easy to use.^25^

Herein we report a similar prevalence of malnutrition than other series,^3^, ^14^, ^26^ small differences might be present because, in our cohort, patients were evaluated before systemic treatment started (or during the first days of therapy), thus, the nutritional impact of treatment‐related side effects were not reflected in this study.

Regarding body composition measurements, during several years, dual energy X‐ray absorptiometry (DXA) was considered as a gold‐standard technique; it measures lean body mass, fat mass, and bone mineral mass. This is a high sensitive, non‐portable method with low radiation exposure, which produces a two‐dimensional image, in consequence, it does not distinguish between subcutaneous and VAT.^27^ Due to these limitations, computerized tomography (CT) and magnetic resonance images (MRI) have emerged as better techniques for evaluating body composition in cancer patients, they allow the measurement of lean body mass, subcutaneous and visceral fat mass.^27^ Despite CT images are available in most patients, it is not routinely measured, and the measurements should be performed by a trained clinician, which limits its routine use in the clinical practice.^28^

In this context, BIVA represents a valuable method. This is an easy‐to‐use, cheap and portable system, which provides information about lean, water, and fat mass.^27^ Specifically, BIA estimates total body water, indirectly it determines LM, assuming a constant hydration factor of 73%; FM is calculated from the weight difference between lean body mass and body weight.^27^ Most nutritional studies in HNC include BIA measurements,^26^, ^29^, ^30^ which has been also combined with other methods including DXA.^31^ BIVA is less frequently used,^32^ it provides a qualitative evaluation of hydration, cell integrity and BCM, contributing with additional information to BIA measures. It has been reported that BIA has good consistency (particularly FFM) in evaluating body composition during treatment in HNC patients.^22^ Remarkably, one study that compared BIA and DXA in HNC undergoing systemic treatment, only found a slight underestimation (without statistical significance) of LM using BIA.^33^ Based on this, the results of body composition measurement in our study, using BIVA are reliable and reinforced by the high number of participants, and the strict protocol for nutritional evaluation.

As BIVA determines several parameters, their value and applicability for diagnosing malnutrition, alone and in combination, was evaluated in our cohort. PA and SPA are matter of interest, as they have been reported in several recent publications. Specifically, a recent study described a PA of 4.42° as the cutoff point that better differentiate patients with and without malnutrition,^34^ in our study, a PA difference of 0.5° was observed between well‐nourished and malnourished patients and a cutoff point of 4.9° (AUC 0.628) was identified. Importantly, PA was the only parameter that significantly changed in patients with HNC, and weight loss independently of their baseline BMI. In some series, PA has been described as the most crucial predictor of survival and a risk factor for death in HNC, specifically, patients with PA < 4.42° had a significant lower survival than patients with PA > 4.42° (19.8 vs. 34.4 months, respectively).^34^ In this line, a previous study in 123 patients reported that a PA cutoff value at 5.95° provided the best prediction of 5‐year survival in HNC.^29^ Some authors have sugested the importance of combining BMI and weight loss for predicting survival in cancer patients, specifically, a study that included 8160 cancer patients, reported a longer survival in patients with stable BMI 25–28 kg/m^2^, while the percentage of weight loss associated with lower categories of BMI were related to shorter survival.^35^ Our study does not include a specific survival analysis, but this evaluation should be further performed. Also, we observed that C‐RP was negatively correlated with PA and SPA inn this cohort, reflecting an association between inflammation and these BIVA‐determined parameters; as previously described, inflammation has been associated with increased mortality.^36^

Interestingly, in our study BCMI was a relevant marker for identifying patients with malnutrition, its use as a single marker (in males) or in combination with BCM and FFMI in females can accurately diagnose malnutrition. Previous studies have reported lower BCMI and FFMI in female patients with cancer at risk of malnutrition compared with males,^37^, ^38^ suggesting that different algorithm diagnosis might be used for diagnosing malnutrition in men and women. Differences might be explained due to sex‐related baseline differences in body composition,^39^ but sex‐related differences have not been associated yet with survival in locally advanced HNC patients.

Despite the elevated number of participants, and the use of BIVA measurements, this study reflects the limitations of using single parameters for diagnosing malnutrition, despite acceptable AUCs, their specificity is limited; it reflects the importance of their combination of different techniques and the relevance of an integrated nutritional evaluation and follow‐up.^15^, ^25^ In this context, and to the best of our knowledge, this is the first study that proposes a comprehensive clinical algorithm for using BIVA‐derived parameters for accurately identifying patients with malnutrition. Notwithstanding, we understand that is not easy to remember and that its applicability in the clinical practice can be limited. Recent studies have reported additional simple techniques for improving nutritional evaluation in HNC, including nutritional ulttrasound,^14^ but their validation should still be performed.

In contrast to other types of cancer, in HNC, there are no reports about fat mass and clinical outcomes, despite it is currently a matter of interest and is being evaluated in combination with muscle mass using CT‐scans.^40^ In this context, in our study, FM > 12 also played a role identifying patients with malnutrition despite increased BCMI and BCM, thus, the impact of fat mass and clinical outcomes including complications and mortality, should be explored in HNC patients.

Regarding biochemical parameters, its evaluation in routinely recommended, despite previous studies have suggested the limitations of visceral proteins in nutritional evaluation.^21^ In this study, albumin and prealbumin only positively correlated with PA and SPA in both sexes.

Finally, a comprehensive nutritional evaluation should also include the perception of the patient. According to our results, PG‐SGA reflects the nutritional status of the patient including muscle mass‐ related parameters, fat mass‐related parameters, PA and SPA. In contrast, the ECOG, only reflects muscle‐mass related parameters, thus, the PG‐SGA provides additional information in the holistic evaluation of the patient, for that reason, the authors suggest its routine use in the clinical practice.

This study has some limitations, this is a cross‐sectional study, thus, complications, and survival were not analysed. Clinical evaluation was performed by multiple clinicians, but a detailed, strict protocol and the same equipment was used un all centers. Other clinical variables that could influence the nutritional status were not evaluated (previous neoplasms, autoimmune diseases). Finally, we only used one criterion (low muscle function) to define sarcopenia while its use in combination with low muscle mass is recommended.^20^ In contrast, this study has several strengths, it includes a large number of participants, with different stages and both sexes. Furthermore, the study protocol included a comprehensive nutritional evaluation that combined anthropometric, functional and biochemical parameters using the same protocol as previously described.

Taken together, our results show a key role of BIVA in the nutritional evaluation of patients with HNC. Not only PA, but also BCMI represents a valuable tool for diagnosing malnutrition, but sex‐specific evaluation is recommended due to sex‐related baseline differences in body composition. Furthermore, BCM has a significant value for diagnosing sarcopenia, while PA and SPA have a relevant role for identifying patients with malnutrition according to the PG‐SGA, which provides a holistic nutritional evaluation of the patient. Further studies that evaluate the relevance of these parameters for predicting clinical outcomes and survival are still necessary.

## Conflict of interest

The authors have no conflicts of interest to declare.

## Funding

This work was funded by FRESENEIUS KABI® and Project PI23/01554 funded by Instituto de Salud Carlos III (ISCIII) and co‐funded by the European Union, JR19/00050, ISCIII. The funders were not involved in the study design, collection, analysis, interpretation of data, the writing of the manuscript, or the decision to submit it for publication.

## Supporting information


**Figure S1.** Supporting Information.

**Figure S2.** Supporting Information.

**Figure S3.** Supporting Information.

**Table S1.** Body composition characteristics of patients with HNC, BMI < 22 Kg/m2 and weight loss using BIVA.

**Table S2.** Body composition characteristics of patients with HNC, BMI 22–25 Kg/m2 and weight loss using BIVA.

**Table S3.** Body composition characteristics of patients with HNC, BMI 25–30 Kg/m2 and weight loss using BIVA.

**Table S4.** Body composition characteristics of patients with HNC, BMI > 30 Kg/m2 and weight loss using BIVA.

**Table S5.** Predictive Value of nutritional assessment methods on malnutrition in patients with head and neck cancer in males and females.

## Data Availability

All data generated or analysed during this study are included in this article. Further enquiries can be directed to the corresponding authors.
