# Supplementary figures and images for: Improving the nutritional evaluation in head neck cancer patients using bioelectrical impedance analysis: Not only the phase angle matters
Source: J Cachexia Sarcopenia Muscle. 2024 Oct 24;15(6):2426–36. doi: 10.1002/jcsm.13577 (PMC11634526; doi:10.1002/jcsm.13577)

## Slide 1
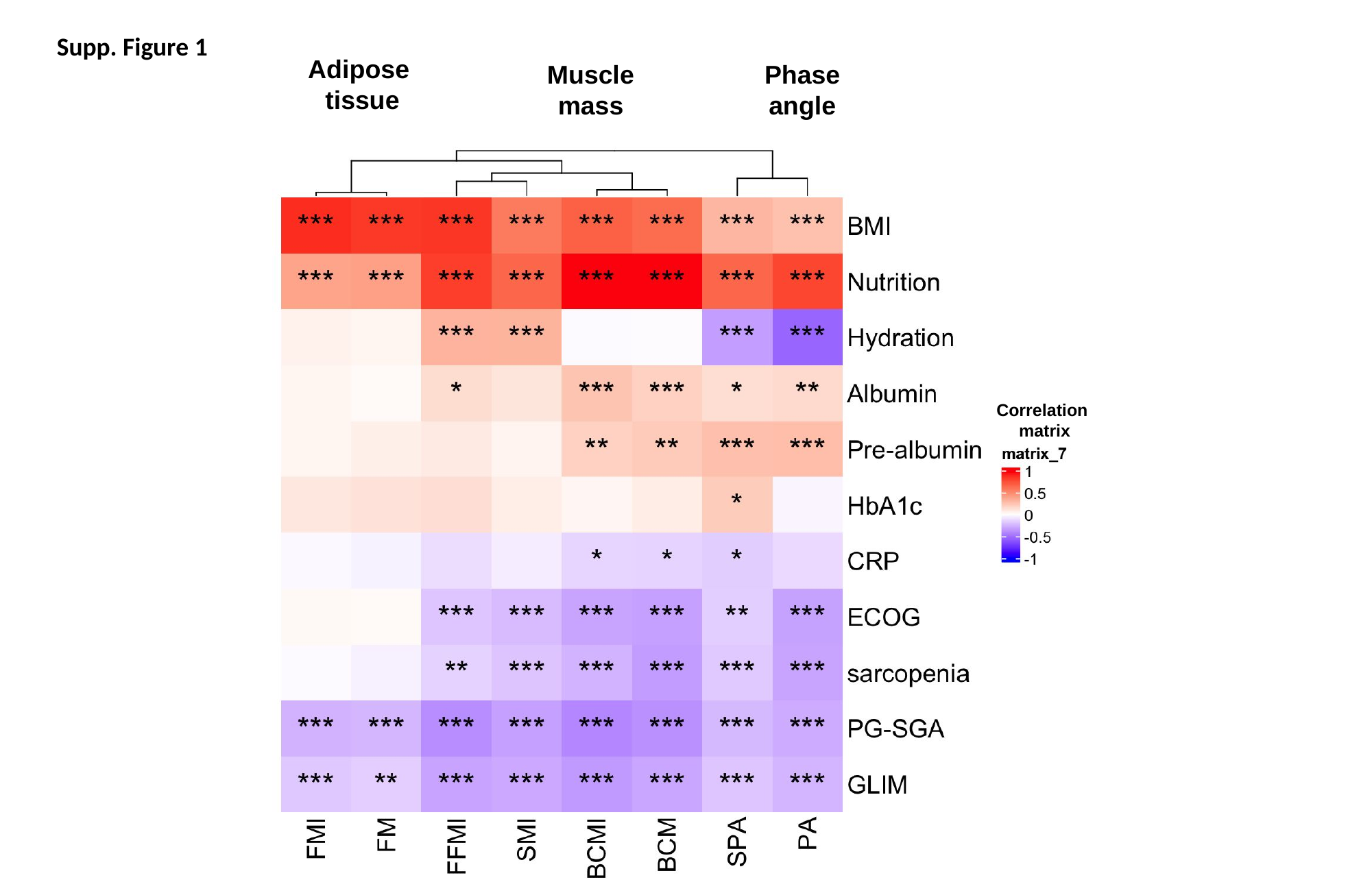

Supp. Figure 1
Adipose
tissue
Muscle
mass
Phase
angle
Correlation
 matrix

Supplement: Supplementary file 1 — Figure S1. Supporting Information. [file JCSM-15-2426-s001.pptx]

## Slide 1
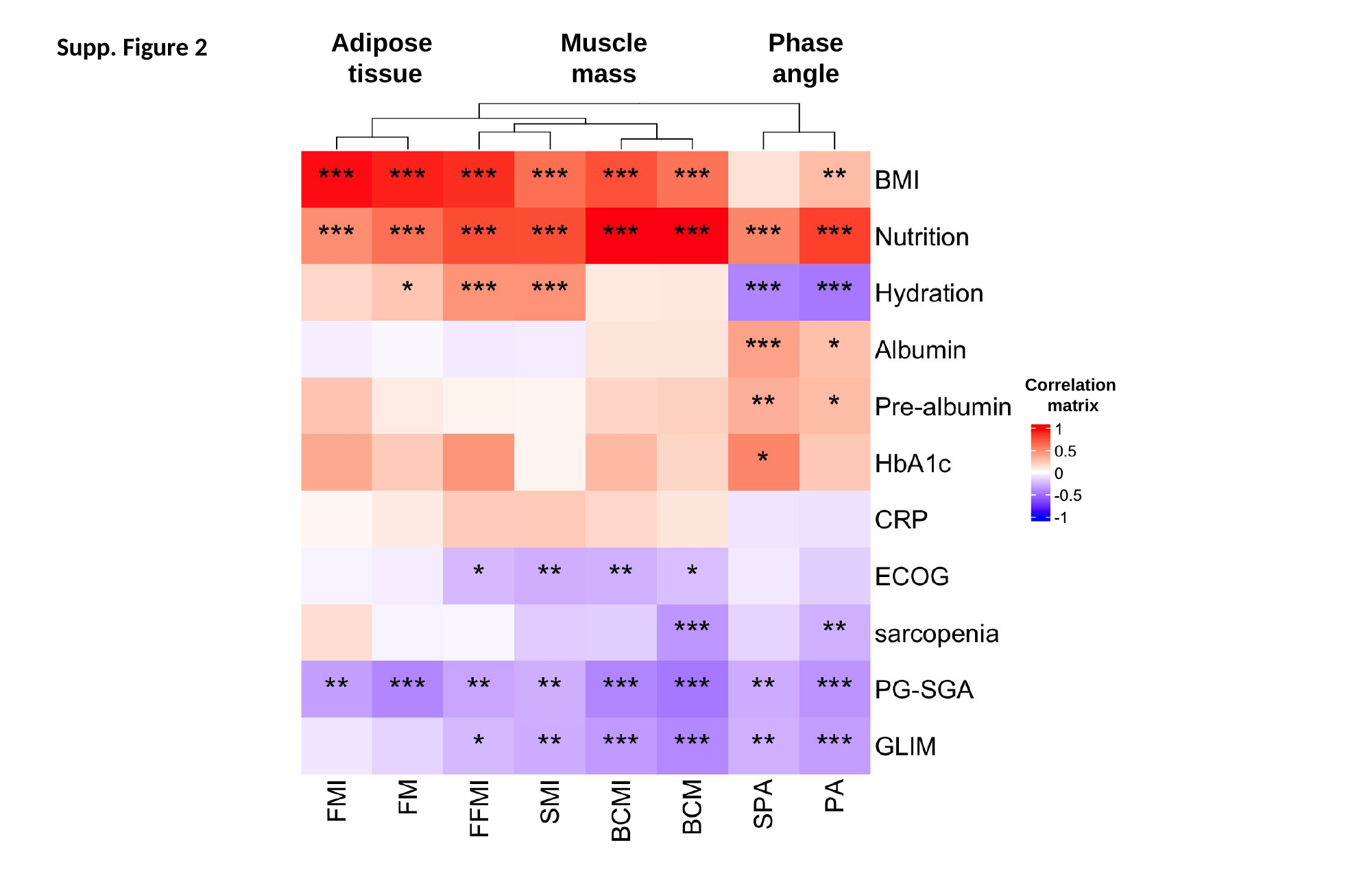

Adipose
tissue
Muscle
mass
Phase
angle
Supp. Figure 2
Correlation
 matrix

Supplement: Supplementary file 2 — Figure S2. Supporting Information. [file JCSM-15-2426-s004.pptx]
