# Supplementary material for: Improving the nutritional evaluation in head neck cancer patients using bioelectrical impedance analysis: Not only the phase angle matters
Source: J Cachexia Sarcopenia Muscle. 2024 Oct 24;15(6):2426–36. doi: 10.1002/jcsm.13577 (PMC11634526; doi:10.1002/jcsm.13577)
Supplement: Supplementary file 3 — Figure S3. Supporting Information. [file JCSM-15-2426-s007.pptx]

## Slide 1
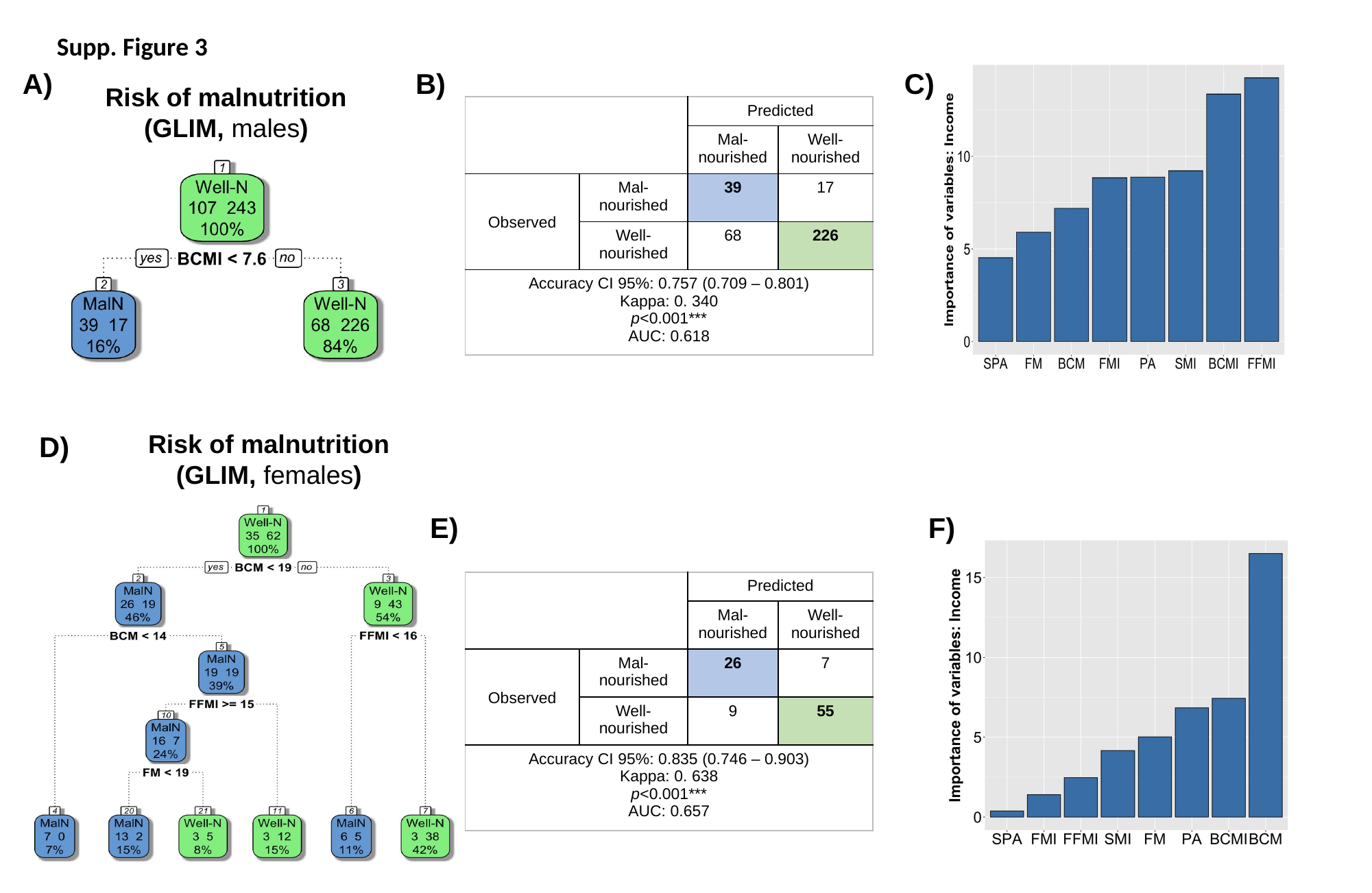

Supp. Figure 3
A)
B)
C)
Risk of malnutrition
(GLIM, males)
| | | Predicted | |
| --- | --- | --- | --- |
| | | Mal-nourished | Well-nourished |
| Observed | Mal-nourished | 39 | 17 |
| | Well-nourished | 68 | 226 |
| Accuracy CI 95%: 0.757 (0.709 – 0.801) Kappa: 0. 340 p<0.001\*\*\* AUC: 0.618 | | | |
B)
Risk of malnutrition
(GLIM, females)
D)
E)
F)
| | | Predicted | |
| --- | --- | --- | --- |
| | | Mal-nourished | Well-nourished |
| Observed | Mal-nourished | 26 | 7 |
| | Well-nourished | 9 | 55 |
| Accuracy CI 95%: 0.835 (0.746 – 0.903) Kappa: 0. 638 p<0.001\*\*\* AUC: 0.657 | | | |
