# Supplementary material for: Improving the nutritional evaluation in head neck cancer patients using bioelectrical impedance analysis: Not only the phase angle matters
Source: J Cachexia Sarcopenia Muscle. 2024 Oct 24;15(6):2426–36. doi: 10.1002/jcsm.13577 (PMC11634526; doi:10.1002/jcsm.13577)
Supplement: Supplementary file 4 — Table S1. Body composition characteristics of patients with HNC, BMI < 22 Kg/m2 and weight loss using BIVA. [file JCSM-15-2426-s008.docx]

**Supplementary Table 1.** Body composition characteristics of patients with HNC, BMI < 22 Kg/m^2^ and weight loss using BIVA

**Weight loss**

|  | **All patients** | **<5 %** | **5-10 %** | **>10%** | ***p*** |
| --- | --- | --- | --- | --- | --- |
|  | ***N=120*** | ***N=32*** | ***N=24*** | ***N=64*** |  |
| Xc | 52.9 (9.42) | 52.1 (10.2) | 56.1 (8.57) | 52.2 (9.20) | 0.207 |
| Rz | 621 (82.6) | 603 (73.2) | 626 (82.1) | 630 (87.4) | 0.324 |
| PA | 4.84 (0.75) | 4.94 (0.76) | 5.15 (0.69) | 4.67 (0.73) | 0.018 |
| SPA | -0.91 (1.29) | -0.78 (1.16) | -0.50 (1.26) | -1.14 (1.34) | 0.102 |
| BCM | 20.8 (4.48) | 22.3 (3.27) | 21.9 (4.24) | 19.6 (4.78) | 0.006 |
| FM | 10.9 (3.97) | 11.6 (4.22) | 11.5 (3.18) | 10.3 (4.07) | 0.231 |
| FFMI | 15.9 (1.50) | 16.3 (1.12) | 16.2 (1.37) | 15.5 (1.63) | 0.029 |
| FMI | 3.92 (1.37) | 4.06 (1.44) | 4.19 (1.15) | 3.76 (1.41) | 0.346 |
| BCMI | 7.42 (1.27) | 7.77 (0.97) | 7.93 (1.09) | 7.06 (1.36) | 0.003 |
| SMI | 7.71 (1.41) | 8.05 (1.19) | 7.82 (1.46) | 7.51 (1.47) | 0.200 |
| MM | 22.4 (5.43) | 24.2 (4.22) | 22.6 (5.78) | 21.4 (5.67) | 0.051 |
| SMM | 22.4 (5.47) | 24.3 (4.25) | 22.6 (5.84) | 21.4 (5.67) | 0.047 |
| ASMM | 16.4 (3.42) | 17.7 (2.52) | 16.8 (3.39) | 15.7 (3.66) | 0.026 |
| FFM | 44.3 (6.81) | 46.9 (5.19) | 44.9 (6.56) | 42.8 (7.26) | 0.016 |
| TBW | 32.4 (5.32) | 34.5 (4.12) | 32.6 (5.02) | 31.3 (5.70) | 0.022 |
| ECW | 16.9 (2.98) | 17.8 (2.76) | 16.3 (2.47) | 16.7 (3.20) | 0.149 |
| ICWpct | 47.6 (5.06) | 48.5 (4.37) | 49.8 (3.71) | 46.4 (5.50) | 0.011 |
| NAK | 1.26 (0.18) | 1.24 (0.18) | 1.17 (0.15) | 1.31 (0.19) | 0.011 |
| Metabolism | 1351 (130) | 1397 (94.7) | 1388 (125) | 1314 (137) | 0.004 |
| Hydration | 73.3 (0.36) | 73.3 (0.40) | 73.2 (0.33) | 73.3 (0.34) | 0.438 |
| Nutrition | 626 (127) | 669 (89.2) | 664 (123) | 591 (135) | 0.005 |
