# Supplementary material for: Improving the nutritional evaluation in head neck cancer patients using bioelectrical impedance analysis: Not only the phase angle matters
Source: J Cachexia Sarcopenia Muscle. 2024 Oct 24;15(6):2426–36. doi: 10.1002/jcsm.13577 (PMC11634526; doi:10.1002/jcsm.13577)
Supplement: Supplementary file 5 — Table S2. Body composition characteristics of patients with HNC, BMI 22–25 Kg/m2 and weight loss using BIVA. [file JCSM-15-2426-s006.docx]

**Supplementary Table 2.** Body composition characteristics of patients with HNC, BMI 22-25 Kg/m^2^ and weight loss using BIVA

**Weight loss**

|  | **All patients** | **<5** | **5-10** | **>10** | ***p*** |
| --- | --- | --- | --- | --- | --- |
|  | ***N=111*** | ***N=55*** | ***N=35*** | ***N=21*** |  |
| Xc | 51.5 (10.1) | 53.5 (8.69) | 50.5 (11.4) | 48.0 (10.3) | 0.088 |
| Rz | 573 (78.3) | 565 (64.4) | 566 (94.2) | 602 (78.8) | 0.167 |
| PA | 5.22 (0.92) | 5.50 (0.84) | 5.11 (0.87) | 4.72 (0.99) | 0.003 |
| SPA | -0.43 (1.21) | -0.41 (1.14) | -0.36 (1.33) | -0.60 (1.22) | 0.771 |
| BCM | 24.4 (4.80) | 25.8 (4.28) | 23.9 (4.92) | 21.3 (4.48) | 0.001 |
| FM | 15.9 (4.27) | 15.4 (4.36) | 15.9 (4.48) | 17.0 (3.62) | 0.347 |
| FFMI | 17.7 (1.42) | 17.9 (1.27) | 17.7 (1.70) | 17.1 (1.16) | 0.075 |
| FMI | 5.72 (1.42) | 5.44 (1.36) | 5.80 (1.55) | 6.31 (1.20) | 0.050 |
| BCMI | 8.73 (1.39) | 9.14 (1.30) | 8.64 (1.39) | 7.86 (1.22) | 0.001 |
| SMI | 8.41 (1.27) | 8.70 (1.15) | 8.32 (1.26) | 7.77 (1.41) | 0.013 |
| MM | 25.2 (6.11) | 26.4 (5.54) | 25.1 (6.64) | 22.3 (5.91) | 0.031 |
| SMM | 25.2 (6.11) | 26.4 (5.54) | 25.1 (6.64) | 22.3 (5.91) | 0.031 |
| ASMM | 18.5 (3.10) | 19.3 (2.60) | 18.3 (3.38) | 16.8 (3.21) | 0.005 |
| FFM | 49.3 (6.48) | 50.6 (5.47) | 49.1 (7.12) | 46.0 (6.88) | 0.018 |
| TBW | 36.2 (5.12) | 37.0 (4.15) | 36.3 (5.88) | 34.2 (5.74) | 0.104 |
| ECW | 18.0 (2.96) | 17.8 (2.40) | 18.3 (3.14) | 18.2 (3.91) | 0.724 |
| ICWpct | 49.8 (6.08) | 51.7 (4.53) | 49.4 (4.94) | 45.4 (8.66) | <0.001 |
| NAK | 1.14 (0.17) | 1.11 (0.15) | 1.15 (0.18) | 1.24 (0.20) | 0.030 |
| Metabolism | 1457 (140) | 1499 (124) | 1444 (145) | 1368 (130) | 0.001 |
| Hydration | 73.3 (0.32) | 73.3 (0.30) | 73.3 (0.35) | 73.3 (0.34) | 0.713 |
| Nutrition | 744 (139) | 788 (123) | 730 (142) | 653 (129) | <0.001 |
