# Supplementary material for: Improving the nutritional evaluation in head neck cancer patients using bioelectrical impedance analysis: Not only the phase angle matters
Source: J Cachexia Sarcopenia Muscle. 2024 Oct 24;15(6):2426–36. doi: 10.1002/jcsm.13577 (PMC11634526; doi:10.1002/jcsm.13577)
Supplement: Supplementary file 6 — Table S3. Body composition characteristics of patients with HNC, BMI 25–30 Kg/m2 and weight loss using BIVA. [file JCSM-15-2426-s002.docx]

**Supplementary Table 3.** Body composition characteristics of patients with HNC, BMI 25-30 Kg/m^2^ and weight loss using BIVA

**Weight loss**

|  | **All patients** | **<5 %** | **5-10 %** | **>10%** | ***p*** |
| --- | --- | --- | --- | --- | --- |
|  | ***N=165*** | ***N=108*** | ***N=33*** | ***N=24*** |  |
| Xc | 48.6 (10.1) | 48.8 (9.27) | 51.3 (12.7) | 43.8 (7.72) | 0.021 |
| Rz | 523 (68.2) | 520 (68.7) | 530 (68.9) | 531 (67.0) | 0.650 |
| PA | 5.33 (0.93) | 5.39 (0.84) | 5.53 (1.14) | 4.79 (0.81) | 0.007 |
| SPA | -0.41 (1.15) | -0.25 (1.11) | -0.53 (1.30) | -0.88 (0.97) | 0.048 |
| BCM | 27.2 (5.22) | 27.5 (5.01) | 27.9 (5.41) | 24.8 (5.44) | 0.049 |
| FM | 21.9 (4.86) | 21.6 (4.51) | 22.0 (5.31) | 22.6 (5.76) | 0.672 |
| FFMI | 19.3 (1.56) | 19.4 (1.64) | 19.3 (1.42) | 19.1 (1.40) | 0.631 |
| FMI | 7.83 (1.63) | 7.76 (1.61) | 7.73 (1.62) | 8.23 (1.74) | 0.422 |
| BCMI | 9.65 (1.46) | 9.75 (1.38) | 9.80 (1.55) | 9.03 (1.58) | 0.078 |
| SMI | 9.03 (1.34) | 9.08 (1.36) | 9.12 (1.29) | 8.67 (1.33) | 0.372 |
| MM | 27.2 (6.04) | 27.1 (5.87) | 28.2 (6.75) | 26.5 (5.92) | 0.569 |
| SMM | 27.3 (6.10) | 27.2 (5.95) | 28.2 (6.75) | 26.5 (5.92) | 0.579 |
| ASMM | 20.5 (3.54) | 20.6 (3.43) | 21.0 (3.58) | 19.4 (3.84) | 0.204 |
| FFM | 54.6 (7.62) | 55.0 (7.55) | 54.9 (7.61) | 52.3 (7.84) | 0.300 |
| TBW | 40.3 (6.14) | 40.5 (5.95) | 40.7 (6.51) | 39.0 (6.50) | 0.505 |
| ECW | 19.7 (3.17) | 19.8 (3.11) | 19.1 (3.08) | 19.8 (3.61) | 0.546 |
| ICWpct | 50.5 (5.07) | 50.8 (4.48) | 51.3 (5.92) | 48.1 (5.71) | 0.037 |
| NAK | 1.09 (0.19) | 1.08 (0.17) | 1.07 (0.22) | 1.14 (0.20) | 0.346 |
| Metabolism | 1537 (153) | 1548 (146) | 1551 (162) | 1470 (158) | 0.064 |
| Hydration | 73.4 (0.34) | 73.4 (0.32) | 73.3 (0.41) | 73.5 (0.26) | 0.055 |
| Nutrition | 825 (149) | 834 (146) | 847 (148) | 756 (149) | 0.044 |
