# Supplementary material for: Improving the nutritional evaluation in head neck cancer patients using bioelectrical impedance analysis: Not only the phase angle matters
Source: J Cachexia Sarcopenia Muscle. 2024 Oct 24;15(6):2426–36. doi: 10.1002/jcsm.13577 (PMC11634526; doi:10.1002/jcsm.13577)
Supplement: Supplementary file 7 — Table S4. Body composition characteristics of patients with HNC, BMI > 30 Kg/m2 and weight loss using BIVA. [file JCSM-15-2426-s005.docx]

**Supplementary Table 4.** Body composition characteristics of patients with HNC, BMI >30 Kg/m^2^ and weight loss using BIVA

**Weight loss**

|  | **All patients** | **<5 %** | **5-10%** | **>10%** | **p** |
| --- | --- | --- | --- | --- | --- |
|  | ***N=71*** | ***N=46*** | ***N=16*** | ***N=9*** |  |
| Xc | 47.3 (8.15) | 47.5 (7.98) | 47.8 (9.58) | 45.0 (6.81) | 0.714 |
| Rz | 476 (63.9) | 465 (48.5) | 506 (92.8) | 484 (66.9) | 0.090 |
| PA | 5.58 (0.86) | 5.79 (0.71) | 5.29 (0.91) | 5.03 (1.12) | 0.015 |
| SPA | -0.17 (0.59) | -0.07 (0.55) | -0.19 (0.48) | -0.57 (0.82) | 0.071 |
| BCM | 31.1 (5.46) | 32.2 (4.23) | 29.1 (7.38) | 28.8 (6.07) | 0.059 |
| FM | 30.2 (5.95) | 29.5 (5.80) | 31.1 (6.36) | 32.1 (6.13) | 0.415 |
| FFMI | 21.7 (1.81) | 22.1 (1.52) | 20.5 (2.01) | 21.3 (2.11) | 0.011 |
| FMI | 10.5 (1.85) | 10.2 (1.70) | 10.9 (2.15) | 11.3 (1.88) | 0.193 |
| BCMI | 11.2 (1.59) | 11.6 (1.27) | 10.6 (1.73) | 10.3 (2.26) | 0.016 |
| SMI | 9.91 (1.35) | 10.2 (1.08) | 9.05 (1.65) | 9.86 (1.49) | 0.013 |
| MM | 28.7 (4.44) | 28.9 (4.09) | 27.0 (5.65) | 30.0 (4.27) | 0.299 |
| SMM | 29.0 (4.24) | 29.4 (3.70) | 27.0 (5.65) | 30.0 (4.27) | 0.193 |
| ASMM | 23.2 (3.26) | 23.6 (2.47) | 22.1 (5.05) | 22.9 (2.49) | 0.312 |
| FFM | 60.4 (7.81) | 61.1 (6.36) | 58.0 (11.5) | 61.2 (6.47) | 0.378 |
| TBW | 44.6 (5.60) | 44.9 (4.56) | 43.5 (8.51) | 45.1 (4.75) | 0.696 |
| ECW | 21.1 (3.04) | 21.0 (2.76) | 21.1 (4.00) | 22.0 (2.68) | 0.663 |
| ICWpct | 52.2 (4.49) | 53.2 (3.63) | 51.2 (4.06) | 48.7 (6.99) | 0.010 |
| NAK | 0.96 (0.13) | 0.95 (0.14) | 0.96 (0.14) | 0.98 (0.10) | 0.796 |
| Metabolism | 1657 (152) | 1684 (123) | 1617 (201) | 1584 (176) | 0.104 |
| Hydration | 73.5 (0.36) | 73.5 (0.34) | 73.5 (0.47) | 73.6 (0.24) | 0.852 |
| Nutrition | 961 (157) | 997 (126) | 904 (188) | 880 (193) | 0.033 |
