# Supplementary material for: Improving the nutritional evaluation in head neck cancer patients using bioelectrical impedance analysis: Not only the phase angle matters
Source: J Cachexia Sarcopenia Muscle. 2024 Oct 24;15(6):2426–36. doi: 10.1002/jcsm.13577 (PMC11634526; doi:10.1002/jcsm.13577)
Supplement: Supplementary file 8 — Table S5. Predictive Value of nutritional assessment methods on malnutrition in patients with head and neck cancer in males and females. [file JCSM-15-2426-s003.docx]

**Supplementary Table 5.** **Predictive Value of nutritional assessment methods on malnutrition in patients with head and neck cancer in males and females**

| **Variables** | **Cut-off**▴  **(Sensitivity – specificity)** | **Cut-off**▴  **(Sensitivity – specificity)** |
| --- | --- | --- |
|  | Males | Females |
| Phase angle |  |  |
| PA | 5.5 (0.480 – 0.766)*** | 4.6 (0.768 – 0.538)** |
| SPA | -0.67 (0.530 – 0.660)** | 0.2 (0.618 – 0.676)* |
| Muscle mass |  |  |
| BCM | 22.9 (0.840 – 0.459)*** | 18.9 (0.725 – 0.718)*** |
| BCMI | 8.1 (0.877 – 0.459)*** | 8.0 (0.594 – 0.789)*** |
| FFMI | 17.1 (0.888 – 0.409)*** | 16.8 (0.529 – 0.710)* |
| SMI | 9.0 (0.683 – 0.591)*** | 6.8 (0.544 – 0.718)** |
| Muscle quality |  |  |
| FM | 16.4 (0.669 – 0.567)*** | 18.3 (0.500 – 0.675) |
| FMI | 6.0 (0.660 – 0.594)*** | 5.8 (0.710 – 0.472) |
|  |  |  |

Receiver operating characteristic (ROC) and Cut-off without adjusting for variables for the nutritional assessment methods and the risk of malnutrition in patients with head and neck cancer.

**Abbreviations**: BCM: Body cell mass; BCMI: BCM index; FFMI: Fat-free mass index; FM: Fat mass; FMI: FM index; OR: Odds ratio; PA: Phase angle; SMI: skeletal muscle index; SPA: Standardized PA.
